# High mortality among fishermen along the beaches of lake Victoria: secondary analysis of evidence from a randomized control trial to promote HIV testing and services uptake in Siaya County, Kenya

**DOI:** 10.1186/s12889-025-22830-0

**Published:** 2025-05-02

**Authors:** Kawango Agot, Benard O. Ayieko, Lila A. Sheira, Carol S. Camlin, Jayne Kulzer, Harsha Thirumurthy, Phoebe Olugo, Edwin D. Charlebois, Zachary Kwena

**Affiliations:** 1 Impact Research and Development Organization, Kisumu, Kenya; 2 University of California, San Francisco, Oakland, United States of America; 3 University of Pennsylvania, Philadelphia, United States of America; 4 Kenya Medical Research Institute, P.O Box 614-40100, Kisumu, Kenya

**Keywords:** Fishermen, Kenya, Mortality causes, Lake Victoria

## Abstract

**Introduction:**

Forty years into the epidemic, HIV remains a significant cause of death among migratory populations such as fisherfolk. Fishermen, in particular, face heightened HIV acquisition risk associated with their high alcohol consumption and engagement in transactional sex. Additionally, the increased risk of other life-threatening conditions among fishermen is often under-recognized. We sought to document incidents and possible causes of death among fishermen on Lake Victoria beaches in Siaya County, Kenya.

**Methods:**

This study reports on deaths among fishermen enrolled in a randomized controlled trial testing whether using a social network-based approach to distribute HIV self-kits with financial incentives compared to counselor-led testing can increase fishermen’s HIV testing, uptake of antiretroviral therapy or pre-exposure prophylaxis following testing, and virologic suppression. Eligible men were aged ≥ 18 years and primarily engaged in the fishing industry. Participants were recruited between July 2020 and February 2022 and followed up for six months post-enrolment to assess the clinical outcomes. We gathered incidents of death from beach leaders, friends, workmates, and family and summed them to compute a crude mortality rate. All cases were reported to the ethics committees of participating institutions and the study’s Data and Safety Monitoring Board.

**Results:**

We screened 1,509 registered fishermen, of whom 934 were mapped to close social networks (the intent to treat sample), and 733 were enrolled. At baseline, participants’ median age was 36 years, 78% were married/cohabiting, 68% attained primary education or below, the majority (57%) earned ≤ USD 83 a month, and all were engaged in fishing/fish-related trade. During the study period, 12 deaths occurred, resulting in a mortality rate of 1,284 per 100,000, 3.1 times higher than that of the general Kenyan male population (419 per 100,000). Primary causes of death included cancers (*n* = 3, 25%), cardiovascular disease (*n* = 3, 25%), HIV-related complications (*n* = 2, 17%), alcohol-related incidents (*n* = 2, 17%), and other causes (*n* = 2, 17%).

**Conclusions:**

The causes of death were varied, underscoring the need for a multi-disease approach to address the health risks in high-risk occupations like fishing. Since HIV is one of several significant health threats to fishermen, efforts to end HIV must also address other life-threatening conditions.

**Supplementary Information:**

The online version contains supplementary material available at 10.1186/s12889-025-22830-0.

## Introduction

The working environment along Lake Victoria exposes fishermen to a higher risk of HIV acquisition [1, 2, 3, 4, 5]. and mortality [6, 7] compared to men in the general population [8]. Heavy alcohol use, funded by disposable fishing income [4, 5], is linked to increased alcohol-triggered violence [6] and exacerbates underlying health conditions [9]. Alcohol misuse also increases raises HIV risk through high-risk sexual behavior and poor adherence and retention in HIV treatment [10, 11, 12]Alcohol-related deaths account for up to 72% of assault-related and 63% of suicides among Lake Victoria fishermen [6]. The extended time spent fishing and long distance to health facilities further complicate HIV treatment adherence [13], and increases the risk of drowning due to adverse weather conditions that fishermen live with constantly [5, 6, 14].

Forty years into the epidemic, HIV remains a significant health threat among fisherfolk. The HIV prevalence among fishermen in the Kenyan counties surrounding Lake Victoria is 23–29% [2, 3], 7–10 times higher than the 3.1% among adult men nationally [15]. *Opemo et al.*, in their study on common causes of mortality among 3058 fishermen in the same region, found high HIV-related deaths, accounting for one-third of the mortality cases [6]. Other less-reported causes include tuberculosis, malaria, ischaemic heart disease, and cancer [16, 17].

There is limited information on the fatalities and the circumstances under which they happen among people working in high-risk environments, such as fishermen [5, 18]. As part of a study among fishermen along Lake Victoria in Kenya to improve the uptake of HIV prevention and treatment services, this short report reviews and syntheses documents to describe incidents, circumstances, and reported causes of death among fishermen participating in a clinical trial. This information is crucial for understanding the broader context of health outcomes and identifying patterns that may influence public health interventions among fishermen. Further, detailed accounts of these circumstances provide insights into social, environmental, and behavioral causes that contribute to mortality, allowing for the design of prevention interventions.

## Methods

This was a serious adverse events review of the ongoing ‘Owete’ study which is a randomized controlled trial to test whether a social network-based approach and financial incentives can increase men’s HIV testing (both self-testing and linkage to facility-based confirmatory blood-based testing), uptake of antiretroviral therapy (ART) and pre-exposure prophylaxis (PrEP) after testing, and virologic suppression (HIV RNA < 400 c/mL) and PrEP adherence (tenofovir levels of ≥ 1500 ng/mL in urine). Details of the study design and procedures of the parent study are reported elsewhere[19]. In brief, the clusters randomized to the control arm distributed valueless coupons for fishermen to go for counselor-led HIV testing or redeem a self-test kit at the nearest public health facility. Eligible men were aged ≥ 18 years, primarily working in the fishing or related industry in one of three beach communities in Siaya County, Kenya. Recruitment of eligible men, stratified by three beaches, began in July 2020 and was completed in February 2022. After the mapping of the beaches, the follow-up period for the intervention was three months, with an additional 3-month period for assessment of clinical outcomes.

Here, we report on deaths that occurred during the period November 2021 through January 2023, gathered from multiple sources, including reports by beach leaders, friends, or workmates, when tracing participants for follow-up appointments. Once a report of death was received, the study team collected additional information from the informants, which we corroborated with family members where applicable, to report to the ethics committees of the sponsoring and implementing institutions and later on to the Data and Safety Monitoring Board for the study. Information collected included probable cause of death from informants and, where appropriate, corroborated information with other sources. Study records were assessed to evaluate known deaths among study participants during the study follow-up period. We share the rate, causes, and description of participant deaths during the implementation of the study. Causes of death were grouped by category based on the primary reported cause of the disease: cardiovascular disease (i.e., stroke, hypertension), HIV (opportunistic infection, ART default), cancers, including alcohol-related incidents, and all others. We calculated the crude mortality rate by dividing the number of deaths that occurred among study participants during the follow-up period (*n* = 12) by the total number of individuals in the cohort (934), then multiplying by 100,000 to standardize to rate. Person-time was measured from the date of the census to the date of death for participants who died or the end of the follow-up period (6 months post-baseline) for those who survived or were lost to follow-up. We standardized the mortality rate by expressing it as the number of deaths per 100,000 person-years to allow for comparison to the mortality rate among Kenyan men.

## Results

We comprehensively mapped social networks among 1,509 registered fishermen working in three purposively selected beach communities along Lake Victoria, Kenya, of whom 934 were mapped to close social networks and 733 enrolled in the study. Table 1 shows the number of participants enrolled in the three beach communities.

**Table 1:** Number of participants enrolled at participating beach communities

Number recruited at each stage	Beach A	Beach B	Beach C	Total
Number registered at the beach	175	503	831	1509
Number mapped to close social network	149	321	464	934
Number enrolled in the study	106	262	365	733

Participants mapped to close social networks were those connected to other fishermen within a defined network/group, and based on this, the members of that network were enrolled in the study. Out of 1509 in the registers at the three beaches, 575 men were excluded because they were not mapped to a close social network, and 201 of 934 mapped to a close social network were excluded for various reasons, including loss to follow up, the cluster being too small, death, and withdrawal. At baseline, participants’ median age was 35.5 years (interquartile range: 30.1, 42.3), 78% were married/cohabiting, 68% attained primary education or below, and the majority (57%) were earning ≤ 10,000 Kenya Shillings a month (1 USD = 120 Shillings). Among the 934 participants in our intent-to-treat sample, we recorded 12 deaths (1.3% of participants) during the study period, a crude mortality rate of 1,284 per 100,000 fishermen. The person-time at risk measured was 1,833.8 person-years among 1509 fishermen. Reported primary causes of death were cancers (*n* = 3), cardiovascular disease (*n* = 3), HIV-related complications (*n* = 2), alcohol-related incidents (*n* = 2), and other causes (*n* = 2). The circumstances of each case are presented in Table 2.

**Table 2:** Causes of death among fishermen in communities along lake Victoria in Siaya County, Kenya

Beach of work/Residence	Reported/Suspected cause	Description/Circumstances
Beach A	Alcoholic coma	The 56-year old participant was found dead by the roadside. A community leader reported that he went on a drinking spree the previous day, then went to buy food but on his way back he blacked out by the roadside. No one got concerned since it was a common occurrence with him. He was found still unconsciousthe following morning, was rushed to a nearby hospital, put on intravenous fluids, but died shortly after.
Beach A	Head injury following alcohol-triggered brawl	The 29-year old participant was having a drink at a local bar with his friends mid-morning when a disagreement ensued and one of his friends hit him at the back of his head with a piece of timber. He collapsed and started bleeding from the mouth and nose, was rushed to a local health facility but died shortly after arrival.
Beach A	Drowning	The 49-year-old participant drowned when he went fishing with his colleagues. The Beach Management Unit (BMU) officials reported that while with his friends fishing deep inside the lake, they noticed that the boat started leaking. Attempts to scoop out the water failed and as the boat began to sink, his colleagues jumped out and swam to the shore. He was unable to swim and drowned.
Beach B	HIV-related complications	The 69-year old participant developed an abdominal problem which led to persistent diarrhoea and loss of weight. He was taken to a health centre, tested for HIV and enrolled into care. He defaulted from taking ARVs and his condition worsened; he was transferred to a higher-level facility and was admitted for about two weeks. He did not improve, was then transferred to the county referral hospital but died one week after hospitalization.
Beach B	Throat Tumor	A family member reported that the participant, a 33-year old, was diagnosed with tuberculosis and stomach ulcers and put on treatment. He developed difficulty in swallowing; the x-ray showed a growth partially blocking the throat. A biopsy was done but the condition worsened and he died while in hospital before the biopsy results came back.
Beach C	Stroke, likely HIV-related	The 55-year old participant, who was known HIV-positive, was reported to have died while receiving treatment at a local hospital. He had collapsed at his place of residence and was rushed to hospital for emergency treatment. He was diagnosed with stroke and urine retention, was catheterized and transferred to a higher level hospital where he died after a few days of hospitalization.
Beach C	Benign Prostatic Hypertrophy and Diabetes	A 75-year old participant who had had diabetes was rushed to the regional hospital in Kisumu with swollen scrotum and urine retention, for which he had a urinary catheter. He was operated on to ease the swelling and urine retention but died while undergoing treatment.
Beach C	HIV-related complications	The 35-year old participant, who was HIV positive and on treatment, was concerned about losing weight and getting weaker over time. He confided in the BMU officials and requested for support from them to go home and get treatment. While at home, his condition worsened and he died.
Beach C	Suspected Penile cancer	The 20-year old participant collapsed at the beach. It was reported that he been suffering from a chronic sexually transmitted infection (had a chronic ulcer on the genital area but was HIV negative) and had been on treatment on and off, potentially for an STI.
Beach C	Liver cancer	The 59-year old participant was admitted at the regional hospital in Kisumu with liver cancer and died while undergoing treatment.
Beach C	Drowning following physical assault	The 39-year old participant was killed during a fight with his colleagues while in the lake. He was physically assaulted and thrown into the deep waters where, unable to swim, he drowned.
Beach C	Stroke	The 49-year old participant developed a mental illness while at the beach and was taken back home by the wife for proper treatment. While under treatment at home he suffered a stroke and later died.

## Discussion

This short report highlights a context of high mortality risk in a population of men working in Kenya’s Lake Victoria shoreline communities. The mortality rate was 1,284 per 100,000 fishermen, 3.1 times that of Kenyan men (419 per 100,000)[20]. HIV disease was linked to at least 17% of the deaths among fishermen in our study, which was conducted in a region with a high burden of HIV disease [2, 3].

Beyond HIV, cancers (including those associated with alcohol use and sexually transmitted infections) and alcohol-related actions were also associated with several deaths. Our study observed deaths related to alcohol-related violence, which corroborates previous research from a large retrospective study among Lake Victoria fishermen where alcohol was the contributing factor to 72% of assault-related deaths [6]. Several other studies have demonstrated that hazardous alcohol use is associated with high-risk behaviour, injuries, and death[18,21–23]. Interventions to reduce alcohol abuse and dependency in this population are urgently needed to reduce violence and indirectly augment HIV intervention efforts and subsequent premature mortality.

The circumstances of these deaths underscore the reality that the risk of HIV for Kenya’s fishermen is not singular but one of many risks these men face in an environment that presents many challenges to men’s health. Men’s livelihood demands and masculine gender norms reinforce a high tolerance for risk-taking. For example, studies report that fishermen downplay HIV risk when compared to what they perceive to be more serious, everyday risks such as drowning and wild animal attacks, especially when staying in the lake for multiple nights in a row [4, 14]. Our findings extend evidence on gender and HIV in Africa that has previously described links between men’s high-risk occupational settings and their risk-taking [1, 2, 4]. Coupled with the risks associated with fishing, men are also poor seekers of health services, who may only seek medical care when close to death, and little could be done to manage their conditions[24,25]. Our team and others have previously reported that clinics are far from men’s workplaces [3], that clinic appointment schedules and wait times are incompatible with fishermen’s busy work schedules [5, 6], and that men in rural East African settings perceive clinics to be spaces for women and children [3]. Notably, it is often men working in occupations that require mobility– frequent movements to and from households and migration to work settings away from residences– who especially face multifaceted barriers to HIV care engagement [7].

We observe several limitations in this analysis. (1) Limited observation time of up to six months, which may be insufficient to observe enough incident deaths to permit meaningful statistical manipulations and make generalized conclusions; (2) We used data from an RCT cohort which, based on inclusion criteria may not be representative of the general population of fishermen and, by extension, other populations outside the fishing communities; (3) We relied on informants to obtain the information on the probable cause of death of the participants. As such, we did not have objective confirmation of the death; (4) We did not have a way of establishing the definite cause of death since we gathered information from informal sources who included BMU leadership and workmates/friends– although we counterchecked the information with family and hospital sources. Despite these limitations, this paper still provides crucial information that is capable of generating debate on what else may be happening in fishing communities with known HIV prevalence rates that may be shadowing everything else.

## Conclusions

Our findings show that deaths in fishing communities are attributed to numerous preventable factors. A suitable intervention may consider engaging men in preventive healthcare convenient for their working hours that ties in with HIV prevention which is a real and salient risk with other prevention activities for CVD and cancer as well as alcohol abuse. Because HIV is just one of many health threats to vulnerable populations of men, there is a need to advance beyond disease-specific, siloed interventions, in favor of multi-disease collaborations to improve the health of communities. Thus, the work to end HIV/AIDS must also involve beginning to end other life-threatening conditions in high HIV prevalence settings.

## Electronic supplementary material

Below is the link to the electronic supplementary material.

Supplementary Material 1

## Data Availability

No datasets were generated or analysed during the current study.

## Abbreviations

ART: Antiretroviral Therapy
PrEP: Pre-exposure Prophylaxis
BMU: Beach Management Unit
