# Supplementary material for: High mortality among fishermen along the beaches of lake Victoria: secondary analysis of evidence from a randomized control trial to promote HIV testing and services uptake in Siaya County, Kenya
Source: BMC Public Health. 2025 May 2;25:1630. doi: 10.1186/s12889-025-22830-0 (PMC12046724; doi:10.1186/s12889-025-22830-0)
Supplement: Supplementary file 1 — Supplementary Material 1 [file 12889_2025_22830_MOESM1_ESM.docx]

**Supplementary Material 1:**

This study was conducted in Siaya county, which borders Lake Victoria and has a population of ~1 million. The county is divided into six sub-counties of Alego Usonga, Gem, Rarieda, Ugenya, Bondo, and Ugunja, with fishing communities in Rarieda and Bondo only. The county has an estimated 79 beaches with approximately 38,000 fisherfolk. The economic activities in the county are mainly fishing and subsistence farming, amidst high rates of poverty[19]. Siaya County is one of the counties in Nyanza region with high rates of HIV coupled with tuberculosis and malaria. HIV prevalence is estimated to be15.3%, with those aged 25-49 years having the highest burden at 28.9%. The highest prevalence is among fisherfolk at 32.1%, explained by high-risk behaviors, including concurrent partnerships, the jaboya sex-for-fish economy, and low condom use, which enhance HIV risks. Most clinics in Siaya County are operated by the Kenya Ministry of Health and typically staffed by clinical officers, and nurses apart from level 4 or higher facilities that may also have medical officers. The United States President’s Emergency for AIDS Relief supports HIV and PrEP services through local implementing partners and integrated within the clinics. The two sub-counties of Rarieda and Bondo bordering Lake Victoria have 70 health facilities offering ART with over 40,000 patients currently in care. Of the 70 clinics, 23 are within 3.5 km of beach communities. The study was conducted in partnership with five of these clinics, three offering 24-hour and weekend services, including HIV care and prevention[19].
